# Capacity for Food of Infants

**Published:** 1891-05

**Authors:** 


### CAPACITY FOR FOOD OF INFANTS.

Dr. H. L. Emmet has recently been making some observations upon this subject, measuring with great care the stomachs of children in autopsies. The conclusions drawn from over one hundred and forty measurements of this sort, are summed up as follows :—

The capacity of an infant's stomach at birth is about one ounce. Increasing in size at the rate of an ounce a month for the first three months, its capacity is then four ounces. After that it increases more slowly, for the next four months at the rate of half an ounce a month. From eight to fourteen months it increases at the rate of a third of an ounce, when the child is a year old attaining a capacity of about eight ounces.

From these facts it will be easy for any one to estimate the probable capacity of a child's stomach at any time from birth to fourteen months of age.

The majority of people use better sense in feeding their dogs and their horses than in feeding their children and themselves. In the New York Hospital for Children, no flesh food is allowed during the first five years of life. It has long been noticed that the children of these institutions are less subject to disease, and recover more quickly when ill, than children who are allowed to eat flesh food. It is also a well known fact that nursing children are much less liable to take disease than older children. Is not this probably due to their simple diet ? A dog or caged lion fed upon so promiscuous a diet as are children in most families, would soon sicken and die. It is no wonder that our children are so puny, and that so large a proportion of them lay in early childhood the foundation for chronic invalidism.

